# Sex-dependent effects of larval food stress on adult performance under semi-natural conditions: only a matter of size?

**DOI:** 10.1007/s00442-017-3903-7

**Published:** 2017-07-06

**Authors:** Elena Rosa, Marjo Saastamoinen

**Affiliations:** Centre of Excellence in Metapopulation Research, Department of Biosciences, University of Helsinki, PO Box 65, Viikinkaari 1, 00014 Helsinki, Finland

**Keywords:** Fitness, Dietary restriction, Resource compensation, Reproductive success

## Abstract

Organisms with complex life-cycles acquire essential nutrients as juveniles, and hence even a short-term food stress during development can impose serious fitness costs apparent in adults. We used the Glanville fritillary butterfly to investigate the effects of larval food stress on adult performance under semi-natural conditions in a population enclosure. We were specifically interested in whether the negative effects observed were due to body mass reduction only or whether additional effects unrelated to pupal mass were evident. The two sexes responded differently to the larval food stress. In females, larval food stress reduced pupal mass and reproductive performance. The reduced reproductive performance was partially mediated by pupal mass reduction. Food stressed females also had reduced within-patch mobility, and this effect was not dependent on pupal mass. Conversely, food stress had no effect on male pupal mass, suggesting a full compensation via prolonged development time. Nonetheless, food stressed males were less likely to sire any eggs, potentially due to changes in their territorial behavior, as indicated by food stress also increasing male within-patch mobility (i.e., patrolling behavior). When males did sire eggs, the offspring number and viability were unaffected by male food stress treatment. Viability was in general higher for offspring sired by lighter males. Our study highlights how compensatory mechanisms after larval food stress can act in a sex-specific manner and that the alteration in body mass is only partially responsible for the reduced adult performance observed.

**Electronic supplementary material:**

The online version of this article (doi:10.1007/s00442-017-3903-7) contains supplementary material, which is available to authorized users.

## Introduction

Nutrient availability is a key factor driving ecological interactions, and any condition causing food limitation is expected to act as an ecological stressor (Fernández-Martínez et al. 2014). However, the nutritive value of a certain diet is meaningful only in the light of the activities performed by the organism of interest, which may vary, for example, in relation to the life stage and between the sexes. Food stress during the sensitive early-life stages can seriously impact juvenile development, growth, and resource acquisition, and subsequently affect a number of adult life-history traits in a negative way. Indeed, negative effects of food stress during development on adult performance, such as on survival, organ functioning, and fecundity, have been reported in several organisms from insects to humans (reviewed by Metcalfe and Monaghan 2001).

A fundamental trait commonly influenced by food stress during development is body size (Blanckenhorn 1999). This response is particularly relevant in organisms with a limited window for growth, such as species with complex life-cycles and those living in seasonally restricted environments. In insects, metamorphosis is followed by a non-growing adult stage and, therefore, the mass achieved prior pupation largely determines adult body size (Nijhout and Williams 1974). The regulation of body size as well as plasticity in body size are under physiological control in insects, and several of the proximate mechanisms that regulate body size can be influenced by larval food stress (Davidowitz et al. 2003; Nijhout 2003; Davidowitz and Nijhout 2004). In the hawkmoth (*Manduca sexta*), for example, growth rate as well as the critical weight (i.e., weight at which growth is not anymore required to initiate metamorphosis) were impacted by larval food stress (Stillwell et al. 2010). However, as body size is often tightly linked to fitness, insects also show numerous adaptive plastic responses to stressful developmental conditions, such as alteration in developmental time, number of developmental stages, and growth rate (Tammaru et al. 2004; Esperk and Tammaru 2010; Teder et al. 2014) to attain a larger body size. Even though often beneficial, such compensatory strategies may also result in costs that are apparent later on in life (Metcalfe and Monaghan 2001; Hou et al. 2011). Additionally, costs of larval food stress may include alterations of metabolic rates and changes in the investment in immune defenses (De Block and Stoks 2008; Saastamoinen and Rantala 2013). Such changes may greatly affect adult performance and fitness, for example, via impacts on lifespan or flight performance (Moret 2000; Therry et al. 2014; Woestmann et al. 2017). Finally, body size is sexually dimorphic in the majority of insects, with females being prevalently larger than males (Stillwell et al. 2010). Females also show greater body size plasticity in insects, especially with respect to manipulation of diet quantity or quality (Stillwell et al. 2010; Walzer and Schausberger 2011). Therefore, sexes may respond differently to the food stress in regard to both compensatory strategies and the resulting effects on adult performance.

Our study has the following aims: (1) to test the impact of short-term larval food stress imposed in the laboratory on adult performance measured under semi-natural conditions, and (2) to assess whether the effects of larval food stress on adult performance are mediated by a reduction in body mass only, or whether additional carry-over effects (i.e. unrelated to body mass reduction) are evident. To achieve these goals we used the Glanville fritillary butterfly as study species. In Finland, the Glanville fritillary butterfly experiences strong seasonality, which both influences resource availability in the wild and reduces the time window for development and reproduction, which set our study on an ecologically relevant context. The last instar larvae are especially vulnerable to starvation, as host plants are often small and scattered in the wild. Laboratory studies have shown that resources acquired during development induce carry-over effects on adult fecundity and lifespan (Saastamoinen et al. 2013a), but it is unknown how strong these effects are under more natural conditions, where compensation via adult feeding may be more constrained than under laboratory conditions. Finally, based on studies conducted under laboratory conditions, there is a suggestion that food stress during development may influence immunity, resting, and flight metabolic rate of adults (Saastamoinen and Rantala 2013), but whether these changes translate to individual performance measures under semi-natural conditions is unknown.

## Materials and methods

### Study species

The Glanville fritillary butterfly (*Melitaea cinxia*) in Finland occurs only in the Åland islands, where it occupies the northernmost limit of the species’ distribution range. In Åland, it has a univoltine life-cycle (i.e., one generation per year), with adult flight season occurring from June to early July. Females emerge with oocytes already present in the ovarioles (Boggs 2003), and lay eggs in clusters of 150–200 on the larval host plant species (*Plantago lanceolata* or *Veronica spicata*). The larvae develop gregariously and overwinter in a compact silk nest (Ojanen et al. 2013). In the spring, the larvae continue feeding gregariously until they reach the 7th and usually final larval instar, at which stage they can become solitary until they pupate. The larvae (*N* = 224) used in the present experiment were collected during the pre-diapause stage from 51 different local populations (i.e., habitat patches) in the municipality of Saltvik in the Åland islands, where only *P. lanceolata* is present as a host plant. The number of families (i.e., number of overwintering nests where the larvae were collected from) per local population varied between one and nine, and the maximum number of individuals collected from one family nest was three.

### Larval rearing and food stress treatment

Most larvae were already in the diapause stage when they were collected from the field. The few larvae that were still about to molt into the diapausing instar were reared in a constant temperature regime with leaves of *P. lanceolata*. Once in the diapause, larvae were kept at +5 °C, with no light until the following spring. The post-diapause larvae were reared in standard laboratory conditions (28:15 °C; 12:12, L/D), mimicking the conditions the black larvae experience in the spring while basking actively in the sun during the day (van Nouhuys and Lei 2004), and fed ad libitum on *P. lanceolata* until the 7th instar.

At the beginning of the 7th instar the larvae were placed in an individual container and assigned to one of two treatments: control or food stress. Different families from the same local population were evenly divided between the two treatments. Larvae were woken up from the diapause in two sets (3 days apart) to ensure that individuals from the two treatment groups would eclose at the same time: the first set of individuals was assigned to the food stress treatment and the second to the control treatment. Previous experiments with similar food stress treatments have shown that the development time is delayed by 2–3 days in the food stress group in comparison to the control group (Saastamoinen et al. 2013a). The larvae in the control group had *P. lanceolata* available ad libitum whereas the larvae in the food stress group were food deprived for 2 days: day two and day four (i.e., 2 × 24 h with 1 day in between), after which they received food ad libitum. All individuals were weighed 1 day after pupation, as pupal mass reliably measures resources acquired during the larval stage. In *M. cinxia* the pupal mass closely relates to the adult mass (0.8 and 0.6 Pearson’s *r* for females and males, respectively; unpublished data from Niitepõld 2010; Niitepõld and Hanski 2013).

### Immunity measurements

To assess immunity, we performed an encapsulation assay on the pupal stage. Encapsulation is a general immune reaction that is activated after the intrusion of metazoan parasites and parasitoids (Pech and Strand 1996). The process involves the recognition of the foreign object, followed by the adhesion of the hemocytes and their aggregation in multiple layers forming a capsule around the parasite, which eventually becomes melanized (Pech and Strand 1996). Higher melanization of the capsule corresponds to higher immune activation. The degree of melanization was measured following methods by Saastamoinen and Rantala (2013) on day three after pupation by inserting a 2 mm nylon (0.18 mm diameter) monofilament on the side of the second-last proleg segment of the pupal cuticle for 1 h at constant room temperature (+27 ± 1 °C). The nylon monofilament was previously rubbed on sandpaper to facilitate the adhesion of the hemocytes, and knots were made at the end of every implant to favor its removal at the end of the measurement. Encapsulated monofilaments were stored at −20 °C immediately after their removal, and photographed from three different angles on white background under a Nikon stereomicroscope. The pictures were analyzed with the software ImageJ (version 1.47) by measuring the melanized area of the monofilament. We calculated the average among the three measurements of the same filament, and subtracted them from the gray value of a blank filament, to obtain increasing numerical values for increasing darkness. Three percent of the pupae did not eclose and 2% had crippled wings as adults. These individuals were omitted from the analyses, while the rest were used in the subsequent life-history assessments.

### Adult performance assessment

Adult life-history traits were assessed in an outdoor population enclosure (32 × 26 × 3 m; e.g. Hanski et al. 2006), which is situated on a dry meadow, and roughly mimics a habitat patch of the butterflies in their natural environment in the Åland Islands. The enclosure is covered with a mesh to prevent the butterflies from escaping. This set-up allows very detailed observations of many key adult life-history traits at the individual level (Hanski et al. 2006). All adults in the present experiment eclosed within a 4-day period in early June. Individuals were marked and sexed 24 h after eclosion, and a total of 100 males and 124 females were released into the enclosure within 48 h of their eclosion. The life-history assessments were performed during 11, at least partially, sunny days, as previous studies have indicated that this approximately corresponds to the lifetime egg production of the females (Saastamoinen 2007). After 11 sunny days the remaining butterflies were collected from the enclosure and the experiment was terminated (see also Duplouy et al. 2013).

We actively searched for mating pairs throughout the enclosure during the sunny periods. Nevertheless, 11% of the matings were missed, based on the number of females that laid eggs even though they had not been observed to mate. The central part of the enclosure contained 150 potted host plants, *P. lanceolata* and *V. spicata*, for female oviposition. We randomized the locations of the plants each day, so that the butterflies would not develop a preference for a particular plant or a particular location. The oviposition site was constantly monitored, the time and location of each oviposition was recorded, and at the end of the oviposition each egg clutch was removed and later on counted to obtain fecundity measures (clutch size and total egg production). Host plants were re-checked each evening and we found eight clutches (2%) that could not be assigned to a mother, and hence were omitted from the data. Number of eggs and larvae sired by a male were determined by combining the observational data about matings and female ovipositions. Previous work by Sarhan and Kokko (2007) has shown that the offspring are sired by the last male a female has mated with.

The enclosure is divided into an 8 × 8 grid, which is systematically censused every second hour (between 9 a.m. and 5 p.m., with each census lasting approximately 1.5 h) to mark the positions of individuals (see Duplouy et al. 2013 for details). The number of grid cells in which a butterfly was recorded throughout the experiment was regressed against the total number of observations of the same butterfly (i.e., the times a butterfly was recorded during the whole experiment), and the residuals of this regression were used as measure of within-patch mobility for each individual (Hanski et al. 2006). We did not census the butterfly locations during completely cloudy periods with low temperature, as the butterflies are not active during such conditions. Previous studies have indicated that early-life mobility (0–3 days) is a particularly relevant trait for females, as after initiating oviposition under this set-up they stay close to the host plants (Hanski et al. 2006). Therefore, we used two different measures in our analyses: early-life mobility (from 0 to 3 days) and total mobility (from 0 to 11 days).

### Statistical analyses

All analyses were conducted with JMP Pro 12 (SAS Institute Inc. 2015). The model selection for all the response variables described below was conducted by backward selection (i.e., removal of the variables associated to the highest non-significant *P*-values from the initial model). Model selection and non-significant values removed are presented in the ESM (Tables A1 and A2, respectively).

#### Developmental traits

The traits examined were development time of the final instar (days), pupal mass, and pupal encapsulation rate. The effects of food treatment on developmental traits were analyzed with a mixed model approach using food treatment, sex and their interaction as fixed factors and population as random factor. To analyze encapsulation rate we added pupal mass as fixed factor and all second order interactions.

#### Reproductive traits

Sexes were analyzed separately. We tested the effect of larval food stress on adult reproduction, and potential contributions of body mass and investment in immunity by including food treatment, pupal mass, encapsulation rate, and the second order interactions with treatment in all models unless stated otherwise. Male or female local population of origin was used as random effect in all mixed models except for clutch size analysis.

A linear model with binomial error distribution was used to analyze the age of a female at her first mating (day 0 or 1), whether a male mated or not, whether a female or male remated or not, and whether the eggs were fertile or not (i.e., larvae hatched from the eggs). To analyze the number of eggs produced in each clutch we used a mixed model for repeated measures with clutch number, male and female treatment, their interaction, their pupal mass, and a factor assessing whether a male or a female had remated before each clutch (i.e. “mating history”) as fixed effects. Male and female IDs were included as random effects to account for repeated measures on the same individual. Finally, a linear mixed model approach was used to analyze the factors affecting the age of a male at his first mating, the age of a female at her first oviposition, and the number of eggs and larvae males or females sired during the experiment.

#### Other adult traits

Additional adult performance traits were mobility (early-life and total) and survival until the end of the experiment. The mobility measures were analyzed with linear mixed models with sex, food treatment, pupal mass, encapsulation rate and all second order interactions with sex and treatment as fixed factors, and population as random effect. Survival based on the daily censuses was analyzed with a parametric approach with Weibull distribution with sex, treatment and their interaction as fixed effects.

Finally, whenever pupal mass but not food stress influenced an adult trait, the same model was repeated without pupal mass to assess whether the effect of food stress was masked by pupal mass.

## Results

### Developmental traits

The 2-day food stress delayed development time in both sexes (*F*_1,212.7_ = 553.2, *P* < 0.0001) and females developed slower than males (*F*_1,213_ = 212.8, *P* < 0.0001; Table 1). The delayed development time due to food stress was more pronounced in males (sex × treatment, *F*_1,214_ = 8.4, *P* = 0.004; Table 1). Larval food stress appeared to reduce pupal mass (*F*_1,207.7_ = 12.4, *P* = 0.0005), but a closer assessment due to a significant interaction between sex and treatment (*F*_1,212.9_ = 4.3, *P* = 0.04) indicated that the effect was only significant in females (Tukey HSD: males, *P* = 0.8; females, *P* = 0.0002). Males weighed less than females as pupae (*F*_1,213.7_ = 322, *P* < 0.0001; Table 1). No effect on pupal encapsulation rate was detected by any of the variables tested (Table A2).

**Table 1:** Averages (±s.e.) for developmental and adult performance traits of females and males in the two larval food treatment groups

	Females		Males	
	Control (*N* = 64)	Food stress (*N* = 60)	Control (*N* = 51)	Food stress (*N* = 49)
*Traits analyzed with pooled data*				
Final instar development time (days)	11.2 (0.1)^a^	13.5 (0.1)^b^	9.2 (0.1)^c^	12.2 (0.1)^d^
Pupal mass (mg)	208.3 (2.1)^a^	195.3 (2.7)^b^	160.6 (2.0)^c^	157.1 (2.1)^c^
Pupal encapsulation rate (mean grey value)	58.6 (0.7)^a^	59.2 (0.9)^a^	58.9 (1.0)^a^	56.6 (1.3)^a^
Early-life mobility (0–3 days)	0.1 (0.1)^a^	−0.1 (0.1)^b^	−0.1 (0.1)^b^	0.1 (0.1)^a^
Total mobility (0–11 days)	−0.7 (0.3)^a^	−0.9 (0.2)^a^	1.0 (0.2)^b^	1.0 (0.2)^b^
Surviving adults recollected at the end (% of initial number)	29.7^a^	30.0^a^	35.3^a^	30.6^a^
*Traits analyzed separately for the two sexes*				
Mated individuals (%)	96.9^a^	98.3^a^	84^A^	75.5^A^
Age at first mating (days)	0.17 (0.05)^a^	0.20 (0.07)^a^	0.93 (0.10)^A^	0.78 (0.14)^A^
Age at first oviposition (days)	1.2 (0.1)^a^	1.6 (0.2)^b^	–	–
Individuals producing any eggs (%)	81.3^a^	90^a^	78.4^A^	53.1^B^
Average total eggs per female (total eggs/number of females)	572 (30.9)^a^	432 (32.7)^b^	726 (63.2)^A^	656 (68.4)^A^
Viable offspring (%)	83.2^a^	81.4^a^	79.5^A^	82.3^A^
Average hatch success per female (total larvae/number of females)	475 (27.2)^a^	359 (30)^b^	596 (57.3)^A^	556 (62.7)^A^

Same superscript letters indicate no significant difference between groups (*P* > 0.07). Upper-case letters refer to males in traits analyzed separately by sex

### Female reproductive traits

A total of 144 matings were recorded during the experiment. Almost 98% of the females released into the population enclosure mated, and more than 80% of them mated on the day of their release. A trend suggested that females with low pupal mass were less likely to mate on the day of their release than heavier ones (♀pupal mass, df = 1, *χ* ^*2*^ = 3.1, *P* = 0.08), with no effect of food stress even after the removal of pupal mass (*P* > 0.7 for all). Twenty-three percent of the females mated more than once. Females that did not remate were either food stressed with low pupal mass or controls with high pupal mass (treatment × pupal mass, df = 1, *χ* ^*2*^ = 5, *P* = 0.03). No main effect due to food treatment was found even after the removal of pupal mass (*P* > 0.5).

**Fig. 1:**
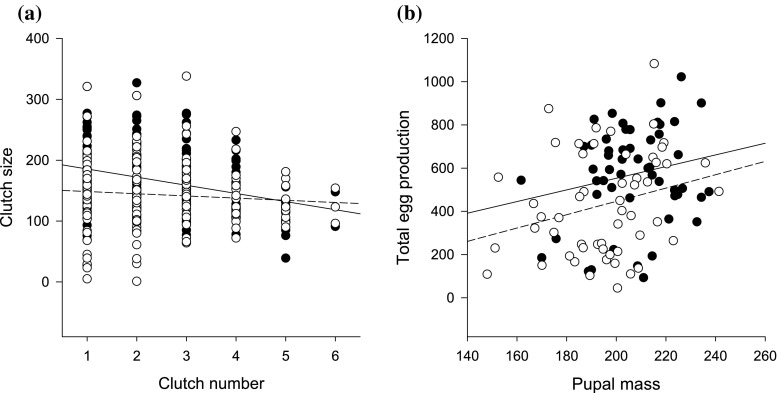
Influence of female clutch number (*P* = 0.0002) and larval food stress (*P* = 0.01) on clutch size (**a**), and effect of female food stress treatment (*P* = 0.02) on total egg production corrected by body mass (**b**). Control treatment is represented by *filled circles* and* solid* and food stress by *open circles* and *dashed line*

Of the mated females, 88% laid at least one fertile clutch of eggs, and none of the variables tested affected egg fertility (Table A2). A trend indicated that females with low pupal mass initiated ovipositing about 1 day later than heavier ones (*F*_1,103.9_ = 3.5, *P* = 0.07), with no effect of food stress even after the removal of pupal mass (*P* > 0.1). The size of egg clutches decreased with increasing clutch number (*F*_1,272.3_ = 14.4, *P* = 0.0002; Fig. 1a). Egg clutches produced by food stressed females were 18% smaller than controls (*F*_1,72.3_ = 6.2, *P* = 0.01; Fig. 1a). Additionally, females with low pupal mass produced smaller egg clutches compared to heavier ones (*F*_1,69.9_ = 8.2, *P* = 0.006). Similarly, the cumulative measures of female reproductive output were reduced by both food stress and low pupal mass (total eggs and total number of larvae produced, *P* < 0.03 for all; Fig. 1b), with no effect of investment in immunity (*P* > 0.4).

### Male reproductive traits

**Fig. 2:**
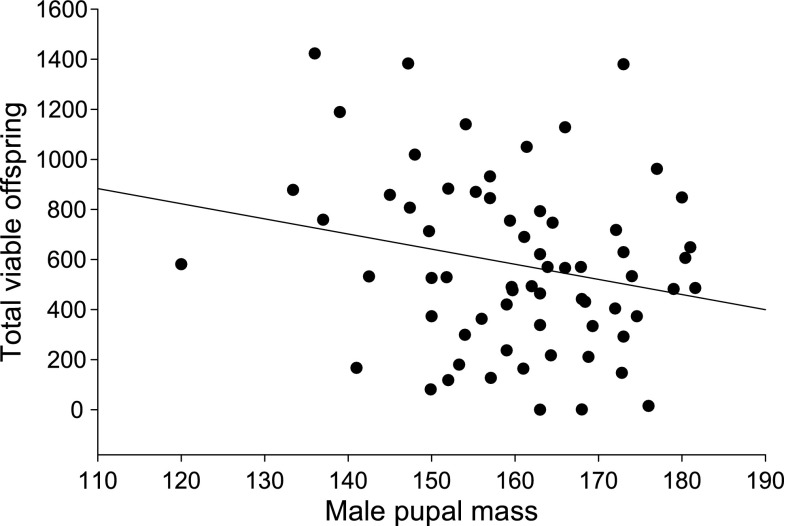
Effect of male body mass on the number of viable offspring sired (*P* = 0.04)

Eighty percent of the males successfully mated, and a trend indicated that food stressed ones were less likely to remate (df = 1, *χ* ^*2*^ = 3.1, *P* = 0.08). Food stressed males and those with low encapsulation rate were less likely to sire any eggs (treatment, df = 1, *χ* ^*2*^ = 10.3, *P* = 0.001; encapsulation, df = 1, *χ* ^*2*^ = 3.9, *P* = 0.03), and the latter was more pronounced in the food stress group (treatment × encapsulation, df = 1, *χ* ^*2*^ = 6.2, *P* = 0.01). Moreover, control males with high pupal mass were also more likely to be sterile (treatment × pupal mass, df = 1, *χ* ^*2*^ = 5.1, *P* = 0.01). The number of eggs sired by fertile males showed a positive tendency with encapsulation rate (*F*_1,49.2_ = 4, *P* = 0.05), which was more pronounced for food stressed ones (treatment × encapsulation, *F*_1,51.6_ = 4, *P* = 0.05). Finally, a trend indicated that larger males sired fewer eggs (*F*_1,52.2_ = 3.1, *P* = 0.08) and significantly fewer larvae (*F*_1,61_ = 4.5, *P* = 0.04; Fig. 2), with no effect of food stress or investment in immunity (*P* > 0.1 for both).

### Mobility and survival

**Fig. 3:**
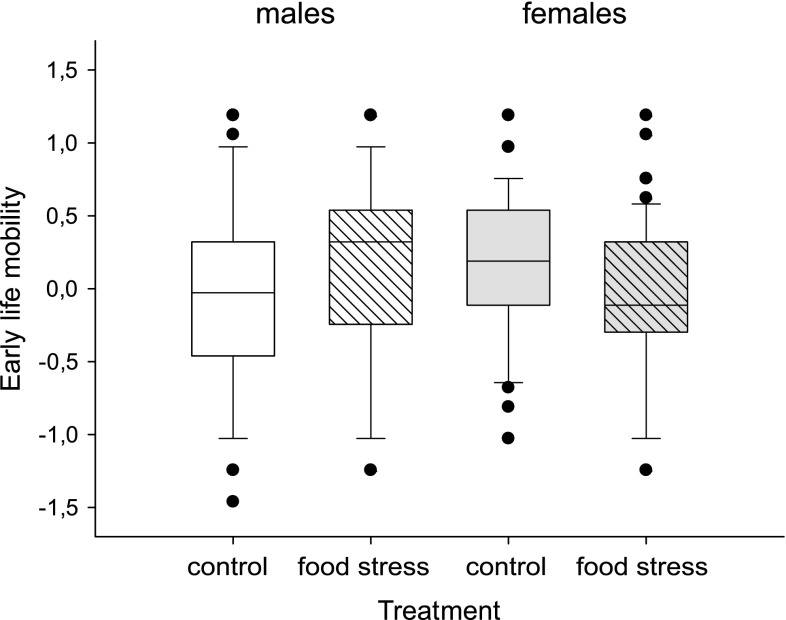
Effect of larval food stress treatment on adult early-life mobility separately for males and females (treatment × sex, *P* = 0.03). Males and females are shown in *white* and *grey fill*, respectively. Food stress treatment is represented by the *striped* pattern

**Fig. 4:**
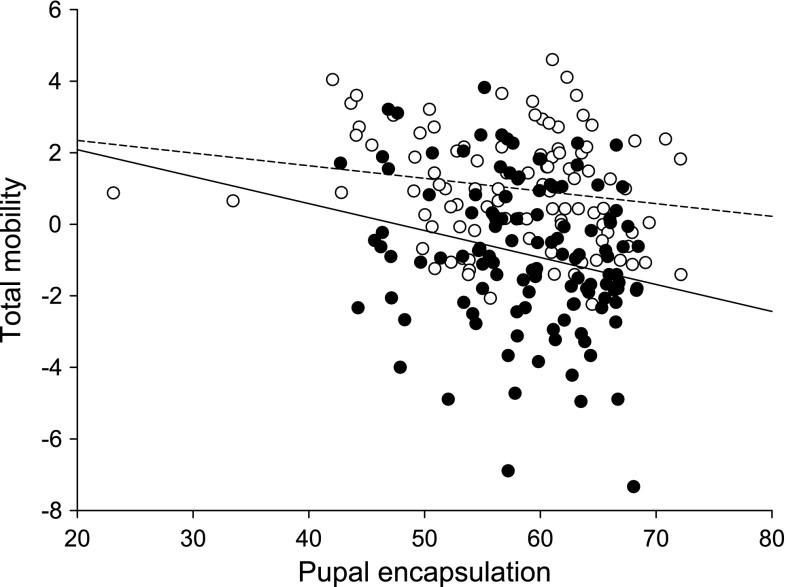
The negative relationship between adult total mobility and pupal encapsulation (*P* = 0.0007) separately for the two sexes (males: *open circles*, *dashed line*; females: *filled circles*, *solid line*)

Food stress had contrasting effects on early-life mobility in males and females (treatment × sex, *F*_1,204.8_ = 4.5, *P* = 0.03; Table 1; Fig. 3): food stressed males were more mobile than controls, whereas in females the opposite was true. In addition, females that had invested more in immunity also had higher early-life mobility, whereas in males the opposite was true (encapsulation × sex, *F*_1,203.4_ = 4.4, *P* = 0.04), with no effect of pupal mass (*P* > 0.1). When considering total mobility, males were more mobile than females (*F*_1,213_ = 50.5, *P* < 0.0001), and individuals that invested less in immunity were more mobile in both sexes (*F*_1,213_ = 11.9, *P* = 0.0007; Fig. 4), with no effect of food treatment or pupal mass (*P* > 0.7 for both). Finally, parametric survival did not differ between sexes or treatments (*P* > 0.09; ESM, Fig. A1). Due to the unusually cold weather in June, the survival rate was higher than in previous similar experiments, as about 30% of the individuals released in the cage were recaptured when the experiment was over (i.e., after 11 sunny days; Table 1).

## Discussion

Several studies on different organisms have assessed responses to food stress during development on adult performance under controlled laboratory conditions (e.g., rats, Hamilton and Bronson 1986; insects, Bauerfeind and Fischer 2005; Boggs and Freeman 2005; Walzer and Schausberger 2011; spiders, Mestre and Bonte 2012). In contrast, responses to food stress under more natural settings are rarer. Studies under semi-natural conditions are important, as ad libitum adult feeding in the laboratory may allow further compensatory responses that cannot as easily take place with naturally scattered resources. Furthermore, under more natural conditions the responses due to food stress during development may be apparent on traits that we cannot easily assess under controlled conditions. Examples of such traits include those related to individual behaviors such as territoriality (Metcalfe and Monaghan 2001).

**Fig. 5:**
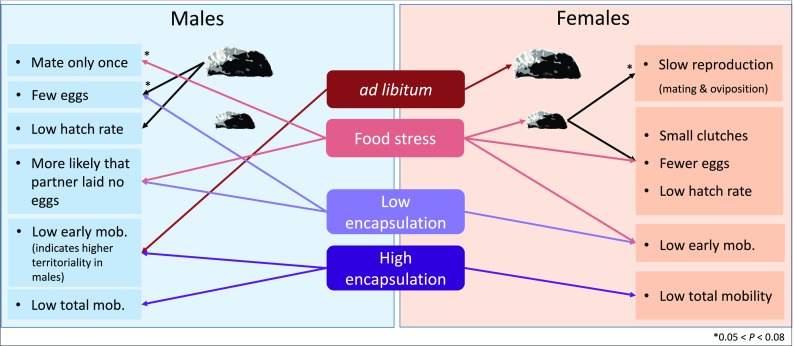
Summary of direct and carry-over effects of food stress on adult performance in males and females. Only main effects are shown for male and female reproductive traits. Refer to the text for interactions

Here we show that even a short, 2-day, larval food stress led to a cascade of effects in the Glanville fritillary butterfly under semi-natural conditions. Many of the observed responses seem mediated via changes in body mass (pupal mass) due to the larval food stress, but additional carry-over effects, independent of body mass, are also apparent. The two sexes show very different responses to the larval food stress, both in regard to developmental and adult performance traits (see Fig. 5 for an overview). Females delayed their development by approximately the same number of days that they experienced food stress for, which is consistent with our previous work (Saastamoinen et al. 2013a). Studies in other butterflies have also shown similar delays in development time (Bauerfeind and Fischer 2009; Tammaru et al. 2015; Saastamoinen et al. 2010). However, this prolonged development did not allow a full compensation in regard to body mass, as food stressed females still metamorphosed at a significantly lower body weight compared with controls, indicating an immediate cost of larval food stress. Conversely, food stressed males delayed their development by 1 day longer than females, which resulted in full compensation in terms of body mass, as food stressed males reached the same weight at metamorphosis as controls. This more extensive prolonging of development time in response to larval stress in males is somewhat surprising based on the general life-histories of butterflies, where males often eclose earlier even with a cost of reduced body mass (i.e., selection for protandry, Wiklund and Fagerström 1977; Singer 1982; Blanckenhorn et al. 2007).

In females, smaller pupal mass led to reduced adult performance, as suggested by an indication that lighter females initiated reproduction later than heavier ones, both in terms of mating and oviposition. In agreement with general findings in insects, lighter females also had lower fecundity (Honěk 1993) in terms of clutch size, total number of eggs and offspring viability (see also below the direct food treatment effects on these traits). Contrarily to the results in females, the cost of small pupal mass is not apparent in males. In fact, lighter males showed somewhat better adult performance than heavier ones, based on an indication that they sired more eggs and on the significantly higher number of viable offspring they produced. This result is contradictory with those on other butterfly species, where larger males produce larger ejaculates and are sexually selected via both female choice and male–male competition (Wiklund and Forsberg 1991). However, in a recent study with the Glanville fritillary butterfly, where adult resources were constrained, resource limited males also sired more offspring (Woestmann and Saastamoinen, in preparation). Moreover, male spermatophore size does not correlate with female reproductive outcome in this species (Duplouy et al. 2017). Consistently with our findings, mating with a smaller male increased female fitness in *Drosophila melanogaster* (Pitnick 1991). Larger *Drosophila* males were more likely to mate with multiple females than smaller males, and subsequently transferred smaller amounts of ejaculate per female. Smaller males, hence, maximized the reproductive effort of a single mating with a larger amount of ejaculate (Pitnick 1991). A similar explanation could potentially underlie our results, but observations of fewer matings obtained by lighter males may have been masked by the slightly female-biased sex ratio and the synchronized release of individuals into the enclosure. This may have relaxed the competition for the possibly non-dominant males (lighter ones in our case), allowing them to mate with an equal number of females as heavier males (i.e., we did not observe male pupal mass influencing his likelihood of mating multiple times; e.g. Grant et al. 1995). Even though this hypothesis is also supported by the high male mating success in the present study (~80%), further studies are required to confirm it.

We were particularly interested to assess whether additional carry-over effects due to larval food stress, but independent of body mass, would be apparent. Females that had experienced food stress during their development had reduced fecundity, as they laid smaller clutches, had lower total number of eggs, as well as eggs with lower hatching rate. These results highlight the importance of larval derived resources on several reproductive performance traits in the Glanville fritillary butterfly, similarly to findings in some other species (Fischer et al. 2004; Bauerfeind and Fischer 2005). The reduction in adult fitness due to larval food stress under semi-natural conditions in the present study were considerably larger than those observed under laboratory conditions (18 and 10%, respectively; Saastamoinen et al. 2013a, b). This likely reflects the harsher conditions females encounter under the semi-natural conditions than in the laboratory, as life-history theory predicts trade-offs to be more apparent under more stressful environments (Hoffmann and Hercus 2000). Males that had experienced food stress during their development also had reduced reproductive performance, as there was an indication that they were less likely to mate multiple times and as they were more likely to sire no offspring. This was the case despite the fact that they reached the same pupal mass as the controls. This result may indicate a potential cost of catch-up growth, in line with various similar effects reported in other organisms (Metcalfe and Monaghan 2001; Hou et al. 2011). Whether the reduced reproductive performance of males is due to their lower sperm quality or quantity (Wedell 2005), or whether food stressed males just mated with females initially of poorer quality remains unknown. Moreover, males that had experienced food stress also had increased early-mobility within the enclosure. This may indicate that food stressed males were less competitive in establishing mating territories and were forced to become patrollers (i.e., fly across the landscape and search for females; Scott 1974; Wahlberg 2000). In contrast, perching (i.e. holding a territory; Scott 1974) is thought to be the most common mate location behavior in *M. cinxia* (Wahlberg 2000; Niitepõld et al. 2011). Food stress in early life stages has also been shown to influence territorial behavior or dominance rank in other organisms, such as fish and birds (Metcalfe and Monaghan 2001).

Larval food stress did not influence investment in immune defense in either sex. Nevertheless, we observed a relationship between immunity and mobility. In females, early-life mobility correlated with higher immune defense, whereas the more sedentary and hence territorial males showed higher investment in immunity. Previous studies have shown that flight can induce an immune response potentially as a general stress response (Saastamoinen and Rantala 2013; Woestmann et al. 2017), but our results indicate that the relationship between immune defense and mobility may be more complex than previously assumed. Especially when considering the mobility during the entire experiment, we observed a trade-off with pupal encapsulation rate in both sexes. It may hence be that individuals with higher investment in immunity are initially able to be more active, but overtime pay a price of this immune investment, which then results in lower total mobility. Finally, consistently with previous laboratory experiments, food stressed individuals did not suffer from reduced adult survival also under semi-natural conditions (Saastamoinen et al. 2013a, b; Saastamoinen and Rantala 2013). This suggests that reserves for body maintenance are likely to come from adult feeding, and that food stressed individuals are able to obtain these resources as effectively as the controls, even under the semi-natural conditions.

One contradictory result of our experiment to previous work relates to the initial developmental responses to food stress. In a previous study with identical larval food stress none of the sexes showed reduced pupal mass when food deprived (Saastamoinen et al. 2013a), whereas here we found that females did show reduced pupal mass. We do not know what underlies this within-species variation in compensatory strategies in response to an identical food stress treatment. However, part of the variation may be explained by initial differences among individuals due to their genetic background (Nijhout 2003), their condition induced by environmental fluctuation or resource availability experienced during the pre-diapause stage (Kingsolver et al. 2009; Saastamoinen et al. 2013b), or even those experienced by their parents. Saastamoinen et al. (2013a) showed how maternal experience of larval stress impacts the way the offspring cope with similar types of stress. More detailed studies assessing the proximate mechanisms (e.g. Davidowitz et al. 2003; Nijhout 2003) underlying the regulation of body size in the Glanville fritillary would be useful to answer these questions in the future. Such studies could also help us to understand the reaction norms defining size at metamorphosis, hence explaining why the food stressed females did not prolong their development time more to obtain a larger pupal mass, even though a large body mass clearly benefits them in terms of fitness.

## Electronic supplementary material

Below is the link to the electronic supplementary material.

Supplementary material 1 (DOC 330 kb)
