# Supplementary material for: Sex-dependent effects of larval food stress on adult performance under semi-natural conditions: only a matter of size?
Source: Oecologia. 2017 Jul 6;184(3):633–42. doi: 10.1007/s00442-017-3903-7 (PMC5511311; doi:10.1007/s00442-017-3903-7)
Supplement: Supplementary file 1 — Supplementary material 1 (DOC 330 kb) [file 442_2017_3903_MOESM1_ESM.doc]

Electronic Supplementary Material

**Table A1.** Final models (i.e. the ones including significant variables) for (A) developmental, (B) female reproductive, (C) male reproductive, and (D) other adult performance traits. Model selection was carried out based on stepwise backward selection (i.e. removal of non-significant variables). For non-significant variables that were initially included in the models, refer to table A2.

| 1. **DEVELOPMENTAL TRAITS** | | | | | | | | | | | | | | | | | | | | | | | |
| --- | --- | --- | --- | --- | --- | --- | --- | --- | --- | --- | --- | --- | --- | --- | --- | --- | --- | --- | --- | --- | --- | --- | --- |
| **Larval development time** | | | | | | | | | | | | | | | | | | | | | | | |
|  | |  | | | | | | | | | | | |  | | | | | | |  | | |
|  | | *Nparm* | | *DFNum* | | | | *DFDen* | | | | | | *F Ratio* | | | | | | | *Prob > F* | | |
| treatment | | 1 | | 1 | | | | 212.7 | | | | | | 553.2 | | | | | | | <.0001 | | |
| sex | | 1 | | 1 | | | | 213 | | | | | | 212.8 | | | | | | | <.0001 | | |
| treatment*sex | | 1 | | 1 | | | | 214 | | | | | | 8.4 | | | | | | | 0.004 | | |
|  | |  | |  | | | |  | | | | | |  | | | | | | |  | | |
| **Pupal weight** | | | | | | | | | | | | | | | | | | | | | | | |
|  | | *Nparm* | | *DFNum* | | | | *DFDen* | | | | | | *F Ratio* | | | | | | | *Prob > F* | | |
| treatment | | 1 | | 1 | | | | 207.7 | | | | | | 12.4 | | | | | | | 0.0005 | | |
| sex | | 1 | | 1 | | | | 213.7 | | | | | | 322.0 | | | | | | | <.0001 | | |
| treatment*sex | | 1 | | 1 | | | | 212.9 | | | | | | 4.3 | | | | | | | 0.039 | | |
|  | |  | |  | | | |  | | | | | |  | | | | | | |  | | |
| **Encapsulation** | | | | | | | | | | | | | | | | | | | | | | | |
| n.s. | |  | |  | | | |  | | | | | |  | | | | | | |  | | |
| 1. **FEMALE REPRODUCTIVE TRAITS** | | | | | | | | | | | | | | | | | | | | | | | |
| **Age at first mating** | | | | | | | | | | | | | | | | | | | | | | | |
|  | | | | *Nparm* | | | | *DF* | | | | | | *L-R ChiSquare* | | | | | | | *Prob>ChiSq* | | |
| pupal weight | | | | 1 | | | | 1 | | | | | | 3.1 | | | | | | | 0.080 | | |
|  | | | |  | | | |  | | | | | |  | | | | | | |  | | |
| **Number of matings (single vs. multiple)** | | | | | | | | | | | | | | | | | | | | | | | |
|  | | | *Nparm* | | | | | *DF* | | | | | | *L-R ChiSquare* | | | | | | | *Prob>ChiSq* | | |
| treatment | | | 1 | | | | | 1 | | | | | | 1.2 | | | | | | | 0.501 | | |
| pupal weight | | | 1 | | | | | 1 | | | | | | 0.2 | | | | | | | 0.646 | | |
| treatment*pupal weight | | | 1 | | | | | 1 | | | | | | 5.0 | | | | | | | 0.025 | | |
|  | | |  | | | | |  | | | | | |  | | | | | | |  | | |
| **Egg fertility (yes vs. no fertile eggs)** | | | | | | | | | | | | | | | | | | | | | | | |
| n.s. | | |  | | | | |  | | | | | |  | | | | | | |  | | |
|  | | |  | | | | |  | | | | | |  | | | | | | |  | | |
| **Age at first oviposition** | | | | | | | | | | | | | | | | | | | | | | | |
|  | | *Nparm* | | | | | *DFNum* | | | *DFDen* | | | | | | *F Ratio* | | | | | *Prob > F* | | |
| pupal weight | | 1 | | | | | 1 | | | 103.9 | | | | | | 3.5 | | | | | 0.070 | | |
|  | |  | | | | |  | | |  | | | | | |  | | | | |  | | |
| **Clutch size** | | | | | | | | | | | | | | | | | | | | | | | |
|  | | *Nparm* | | | | | *DF* | | | *DFDen* | | | | | | *F Ratio* | | | | | *Prob > F* | | |
| fem. treatment | | 1 | | | | | 1 | | | 72.27 | | | | | | 6.2 | | | | | 0.015 | | |
| fem. pupal weight | | 1 | | | | | 1 | | | 69.85 | | | | | | 8.2 | | | | | 0.006 | | |
| female clutch number | | 1 | | | | | 1 | | | 272.3 | | | | | | 14.4 | | | | | 0.0002 | | |
|  | |  | | | | |  | | |  | | | | | |  | | | | |  | | |
| **Total egg production** | | | | | | | | | | | | | | | | | | | | | | | |
|  | | *Nparm* | | | | | *DFNum* | | | *DFDen* | | | | | | *F Ratio* | | | | | *Prob > F* | | |
| treatment | | 1 | | | | | 1 | | | 102.9 | | | | | | 5.4 | | | | | 0.022 | | |
| pupal weight | | 1 | | | | | 1 | | | 102.9 | | | | | | 5.8 | | | | | 0.018 | | |
|  | |  | | | | |  | | |  | | | | | |  | | | | |  | | |
| **Total larvae production** | | | | | | | | | | | | | | | | | | | | | | | |
| **Final model:** | | *Nparm* | | | | | *DFNum* | | | *DFDen* | | | | | | *F Ratio* | | | | | *Prob > F* | | |
| treatment | | 1 | | | | | 1 | | | 102.9 | | | | | | 4.8 | | | | | 0.031 | | |
| pupal weight | | 1 | | | | | 1 | | | 102.4 | | | | | | 4.6 | | | | | 0.034 | | |
|  | |  | | | | |  | | |  | | | | | |  | | | | |  | | |
| 1. **MALE REPRODUCTIVE TRAITS** | | | | | | | | | | | | | | | | | | | | | | | |
| **Mated (yes vs. no)** | | | | | | | | | | | | | | | | | | | | | | | |
| n.s. | |  | | | |  | | | | | |  | | | | | |  | | | | | |
|  | |  | | | |  | | | | | |  | | | | | |  | | | | | |
| **Age at first mating** | | | | | | | | | | | | | | | | | | | | | | | |
| n.s. | |  | | | |  | | | | | |  | | | | | |  | | | | |  |
|  | |  | | | |  | | | | | |  | | | | | |  | | | | | |
| **Number of matings (single vs. multiple)** | | | | | | | | | | | | | | | | | | | | | | | |
|  | | *Nparm* | | | | *DF* | | | | | | *L-R ChiSquare* | | | | | | *Prob>ChiSq* | | | | | |
| treatment | | 1 | | | | 1 | | | | | | 3.1 | | | | | | 0.080 | | | | | |
|  | |  | | | |  | | | | | |  | | | | | |  | | | | | |
| **Egg fertility (yes vs. no fertile eggs)** | | | | | | | | | | | | | | | | | | | | | | | |
|  | | *Nparm* | | | *DF* | | | | | | *L-R ChiSquare* | | | | | | *Prob>ChiSq* | | | | | | |
| treatment | | 1 | | | 1 | | | | | | 10.3 | | | | | | 0.001 | | | | | | |
| pupal weight | | 1 | | | 1 | | | | | | 1.4 | | | | | | 0.241 | | | | | | |
| encapsulation | | 1 | | | 1 | | | | | | 3.9 | | | | | | 0.047 | | | | | | |
| treatment*pupal weight | | 1 | | | 1 | | | | | | 5.1 | | | | | | 0.023 | | | | | | |
| treatment*encapsulation | | 1 | | | 1 | | | | | | 6.2 | | | | | | 0.013 | | | | | | |
|  | |  | | |  | | | | | |  | | | | | |  | | | | | | |
| **Total eggs sired** | | | | | | | | | | | | | | | | | | | | | | | |
|  | | *Nparm* | | | *DFNum* | | | | *DFDen* | | | | | | *F Ratio* | | | | | *Prob > F* | | | |
| treatment  pupal weight | | 1  1 | | | 1  1 | | | | 57.2  52.2 | | | | | | 0.4  3.2 | | | | | 0.532  0.082 | | | |
| encapsulation | | 1 | | | 1 | | | | 49.2 | | | | | | 4.0 | | | | | 0.052 | | | |
| treatment*encapsulation | | 1 | | | 1 | | | | 51.6 | | | | | | 4.0 | | | | | 0.051 | | | |
|  | |  | | |  | | | |  | | | | | |  | | | | |  | | | |
| **Total larvae sired** | | | | | | | | | | | | | | | | | | | | | | | |
|  | | *Nparm* | | | *DFNum* | | | | *DFDen* | | | | | | *F Ratio* | | | | | *Prob > F* | | | |
| pupal weight | | 1 | | | 1 | | | | 61 | | | | | | 4.5 | | | | | 0.039 | | | |
|  | |  | | |  | | | |  | | | | | |  | | | | |  | | | |
| 1. **OTHER PERFORMANCE TRAITS** | | | | | | | | | | | | | | | | | | | | | | | |
| **Early mobility** | | | | | | | | | | | | | | | | | | | | | | | |
|  | | *Nparm* | | | *DFNum* | | | | *DFDen* | | | | | | *F Ratio* | | | | | *Prob > F* | | | |
| treatment | | 1 | | | 1 | | | | 204.7 | | | | | | 0.1 | | | | | 0.8 | | | |
| sex | | 1 | | | 1 | | | | 184.9 | | | | | | 0.002 | | | | | 1 | | | |
| encapsulation | | 1 | | | 1 | | | | 202 | | | | | | 0.6 | | | | | 0.430 | | | |
| treatment*sex | | 1 | | | 1 | | | | 204.8 | | | | | | 4.5 | | | | | 0.035 | | | |
| sex*encapsulation | | 1 | | | 1 | | | | 203.4 | | | | | | 4.4 | | | | | 0.038 | | | |
|  | |  | | |  | | | |  | | | | | |  | | | | |  | | | |
| **Total mobility** | | | | | | | | | | | | | | | | | | | | | | | |
|  | | *Nparm* | | | *DFNum* | | | | *DFDen* | | | | | | *F Ratio* | | | | | *Prob > F* | | | |
| sex | | 1 | | | 1 | | | | 213 | | | | | | 50.5 | | | | | <.0001 | | | |
| encapsulation | | 1 | | | 1 | | | | 213 | | | | | | 11.9 | | | | | 0.0007 | | | |
|  | |  | | |  | | | |  | | | | | |  | | | | |  | | | |
| **Survival** | | | | | | | | | | | | | | | | | | | | | | | |
| n.s. |  | | | | | |  | | | | | |  | | | | | |  | | | | |
|  |  | | | | | |  | | | | | |  | | | | | |  | | |  | |

**Table A2.** Non-significant variables removed for (A) developmental, (B) female reproductive, (C) male reproductive, and (D) other adult performance traits via stepwise backward selection from the models presented in Table A1. Models from which no variables were removed (i.e. all variables initially included were significant) are shown only in Table A1.

| 1. **DEVELOPMENTAL TRAITS** | | | | | | | | | | | | | | | | | | | | | | |
| --- | --- | --- | --- | --- | --- | --- | --- | --- | --- | --- | --- | --- | --- | --- | --- | --- | --- | --- | --- | --- | --- | --- |
| **Encapsulation** | | | | | | | | | | | | | | | | | | | | | | |
|  | | | *Nparm* | | | *DFNum* | | | *DFDen* | | | | | | | *F Ratio* | | | | *Prob > F* | | |
| treatment*sex | | | 1 | | | 1 | | | 197.5 | | | | | | | 0.01 | | | | 0.922 | | |
| sex*pupal weight | | | 1 | | | 1 | | | 212 | | | | | | | 0.3 | | | | 0.612 | | |
| treatment*pupal weight | | | 1 | | | 1 | | | 210.3 | | | | | | | 1.3 | | | | 0.254 | | |
| treatment | | | 1 | | | 1 | | | 210.7 | | | | | | | 0.02 | | | | 0.897 | | |
| sex | | | 1 | | | 1 | | | 215 | | | | | | | 0.05 | | | | 0.828 | | |
| pupal weight | | | 1 | | | 1 | | | 215.3 | | | | | | | 0.5 | | | | 0.486 | | |
|  | | |  | | |  | | |  | | | | | | |  | | | |  | | |
| 1. **FEMALE REPRODUCTIVE TRAITS** | | | | | | | | | | | | | | | | | | | | | | |
| **Age at first mating** | | | | | | | | | | | | | | | | | | | | | | |
|  | | | | *Nparm* | | *DF* | | | | | *L-R ChiSquare* | | | | | | | | | | *Prob>ChiSq* | |
| treatment*encapsulation | | | | 1 | | 1 | | | | | 0.8 | | | | | | | | | | 0.357 | |
| treatment*pupal weight | | | | 1 | | 1 | | | | | 2.4 | | | | | | | | | | 0.118 | |
| encapsulation | | | | 1 | | 1 | | | | | 0.2 | | | | | | | | | | 0.635 | |
| treatment | | | | 1 | | 1 | | | | | 1.1 | | | | | | | | | | 0.298 | |
|  | | | |  | |  | | | | |  | | | | | | | | | |  | |
| **Number of matings (single vs. multiple)** | | | | | | | | | | | | | | | | | | | | | | |
|  | | | *Nparm* | | | *DF* | | | | | | *L-R ChiSquare* | | | | | | | | | *Prob>ChiSq* | |
| treatment*encapsulation | | | 1 | | | 1 | | | | | | 0.4 | | | | | | | | | 0.524 | |
| encapsulation | | | 1 | | | 1 | | | | | | 1.9 | | | | | | | | | 0.171 | |
|  | | |  | | |  | | | | | |  | | | | | | | | |  | |
| **Egg fertility (yes vs. no fertile eggs)** | | | | | | | | | | | | | | | | | | | | | | |
|  | | | *Nparm* | | | *DF* | | | | | | *L-R ChiSquare* | | | | | | | | | *Prob>ChiSq* | |
| treatment*encapsulation | | | 1 | | | 1 | | | | | | 0.04 | | | | | | | | | 0.837 | |
| treatment*pupal weight | | | 1 | | | 1 | | | | | | 0.6 | | | | | | | | | 0.446 | |
| encapsulation | | | 1 | | | 1 | | | | | | 0.006 | | | | | | | | | 0.939 | |
| pupal weight | | | 1 | | | 1 | | | | | | 0.5 | | | | | | | | | 0.462 | |
| treatment | | | 1 | | | 1 | | | | | | 1.7 | | | | | | | | | 0.197 | |
|  | | |  | | |  | | | | | |  | | | | | | | | |  | |
| **Age at first oviposition** | | | | | | | | | | | | | | | | | | | | | | |
|  | | | *Nparm* | | | *DFNum* | | | *DFDen* | | | | | *F Ratio* | | | | | | | *Prob > F* | |
| treatment*pupal weight | | | 1 | | | 1 | | | 22.9 | | | | | 0.2 | | | | | | | 0.689 | |
| treatment*encapsulation | | | 1 | | | 1 | | | 97.6 | | | | | 0.6 | | | | | | | 0.439 | |
| encapsulation | | | 1 | | | 1 | | | 96.8 | | | | | 0.08 | | | | | | | 0.780 | |
| treatment | | | 1 | | | 1 | | | 102.9 | | | | | 1.4 | | | | | | | 0.237 | |
|  | | |  | | |  | | |  | | | | |  | | | | | | |  | |
| **Clutch size** | | | | | | | | | | | | | | | | | | | | | | |
|  | | | *Nparm* | | | *DF* | | | *DFDen* | | | | | *F Ratio* | | | | | | | *Prob > F* | |
| fem. treatment*male treatment | | | 1 | | | 1 | | | 86.8 | | | | | 0.4 | | | | | | | 0.537 | |
| male mating history | | | 1 | | | 1 | | | 103.8 | | | | | 0.005 | | | | | | | 0.942 | |
| fem. mating history | | | 1 | | | 1 | | | 102 | | | | | 0.06 | | | | | | | 0.813 | |
| male treatment | | | 1 | | | 1 | | | 56.1 | | | | | 0.2 | | | | | | | 0.671 | |
| male pupal weight | | | 1 | | | 1 | | | 49.06 | | | | | 1.1 | | | | | | | 0.291 | |
|  | | |  | | |  | | |  | | | | |  | | | | | | |  | |
| **Total egg production** | | | | | | | | | | | | | | | | | | | | | | |
|  | | | *Nparm* | | | *DFNum* | | | *DFDen* | | | | | *F Ratio* | | | | | | | *Prob > F* | |
| treatment*pupal weight | | | 1 | | | 1 | | | 68 | | | | | 0.02 | | | | | | | 0.903 | |
| treatment*encapsulation | | | 1 | | | 1 | | | 97.9 | | | | | 0.5 | | | | | | | 0.478 | |
| encapsulation | | | 1 | | | 1 | | | 98.7 | | | | | 0.3 | | | | | | | 0.617 | |
|  | | |  | | |  | | |  | | | | |  | | | | | | |  | |
| **Total larvae production** | | | | | | | | | | | | | | | | | | | | | | |
|  | | | *Nparm* | | | *DFNum* | | | *DFDen* | | | | | *F Ratio* | | | | | | | *Prob > F* | |
| treatment*pupal weight | | | 1 | | | 1 | | | 28.6 | | | | | 0.01 | | | | | | | 0.913 | |
| treatment*encapsulation | | | 1 | | | 1 | | | 97.6 | | | | | 0.5 | | | | | | | 0.468 | |
| encapsulation | | | 1 | | | 1 | | | 93.9 | | | | | 0.5 | | | | | | | 0.480 | |
|  | | |  | | |  | | |  | | | | |  | | | | | | |  | |
| 1. **MALE REPRODUCTIVE TRAITS** | | | | | | | | | | | | | | | | | | | | | | |
| **Mated (yes vs. no)** | | | | | | | | | | | | | | | | | | | | | | |
|  | | | | | *Nparm* | | | *DF* | | | | | *L-R ChiSquare* | | | | | | | | *Prob>ChiSq* | |
| treatment*pupal weight | | | | | 1 | | | 1 | | | | | 0.2 | | | | | | | | 0.655 | |
| treatment*encapsulation | | | | | 1 | | | 1 | | | | | 0.6 | | | | | | | | 0.454 | |
| treatment | | | | | 1 | | | 1 | | | | | 1.3 | | | | | | | | 0.256 | |
| pupal weight | | | | | 1 | | | 1 | | | | | 2.1 | | | | | | | | 0.148 | |
| encapsulation | | | | | 1 | | | 1 | | | | | 1.9 | | | | | | | | 0.173 | |
|  | | | | |  | | |  | | | | |  | | | | | | | |  | |
| **Age at first mating** | | | | | | | | | | | | | | | | | | | | | | |
|  | | *Nparm* | | | | *DFNum* | | | *DFDen* | | | | | | | | | *F Ratio* | | | *Prob>F* | |
| treatment*pupal weight | | 1 | | | | 1 | | | 64.8 | | | | | | | | | 0.07 | | | 0.796 | |
| treatment*encapsulation | | 1 | | | | 1 | | | 65.1 | | | | | | | | | 0.08 | | | 0.772 | |
| encapsulation | | 1 | | | | 1 | | | 70.8 | | | | | | | | | 0.3 | | | 0.612 | |
| treatment | | 1 | | | | 1 | | | 74.3 | | | | | | | | | 0.9 | | | 0.348 | |
| pupal weight | | 1 | | | | 1 | | | 62.6 | | | | | | | | | 1.9 | | | 0.2 | |
| **Number of matings (single vs. multiple)** | | | | | | | | | | | | | | | | | | | | | | |
|  | | | | | *Nparm* | | | *DF* | | | | | *L-R ChiSquare* | | | | | | | | *Prob>ChiSq* | |
| treatment*pupal weight | | | | | 1 | | | 1 | | | | | 0.2 | | | | | | | | 0.689 | |
| treatment*encapsulation | | | | | 1 | | | 1 | | | | | 0.08 | | | | | | | | 0.784 | |
| encapsulation | | | | | 1 | | | 1 | | | | | 0.2 | | | | | | | | 0.623 | |
| pupal weight | | | | | 1 | | | 1 | | | | | 0.002 | | | | | | | | 0.961 | |
|  | | | | |  | | |  | | | | |  | | | | | | | |  | |
| **Total eggs sired** | | | | | | | | | | | | | | | | | | | | | | |
|  | *Nparm* | | | | | | *DFNum* | | | *DFDen* | | | | | | | *F Ratio* | | | | | *Prob > F* |
| treatment*pupal weight | 1 | | | | | | 1 | | | 55 | | | | | | | 2.5 | | | | | 0.122 |
|  |  | | | | | |  | | |  | | | | | | |  | | | | |  |
| **Total larvae sired** | | | | | | | | | | | | | | | | | | | | | | |
|  | *Nparm* | | | | | | *DFNum* | | | *DFDen* | | | | | | | *F Ratio* | | | | | *Prob > F* |
| treatment*pupal weight | 1 | | | | | | 1 | | | 54.5 | | | | | | | 1.8 | | | | | 0.182 |
| treatment*encapsulation | 1 | | | | | | 1 | | | 52.5 | | | | | | | 3.0 | | | | | 0.089 |
| treatment | 1 | | | | | | 1 | | | 55.5 | | | | | | | 0.3 | | | | | 0.560 |
| encapsulation | 1 | | | | | | 1 | | | 48 | | | | | | | 2.3 | | | | | 0.134 |
|  |  | | | | | |  | | |  | | | | | | |  | | | | |  |
| 1. **OTHER PERFORMANCE TRAITS** | | | | | | | | | | | | | | | | | | | | | | |
| **Early mobility** | | | | | | | | | | | | | | | | | | | | | | |
|  | *Nparm* | | | | | | *DFNum* | | | *DFDen* | | | | | | | *F Ratio* | | | | | *Prob > F* |
| sex*pupal weight | 1 | | | | | | 1 | | | 200 | | | | | | | 0.06 | | | | | 0.803 |
| treatment*pupal weight | 1 | | | | | | 1 | | | 131.6 | | | | | | | 0.4 | | | | | 0.555 |
| treatment*encapsulation | 1 | | | | | | 1 | | | 199 | | | | | | | 2.4 | | | | | 0.121 |
| pupal weight | 1 | | | | | | 1 | | | 195 | | | | | | | 2.4 | | | | | 0.120 |
|  |  | | | | | |  | | |  | | | | | | |  | | | | |  |
| **Total mobility** | | | | | | | | | | | | | | | | | | | | | | |
|  | *Nparm* | | | | | | *DFNum* | | | *DFDen* | | | | | | | *F Ratio* | | | | | *Prob > F* |
| sex*encapsulation | 1 | | | | | | 1 | | | 204.7 | | | | | | | 0.5 | | | | | 0.477 |
| sex*pupal weight | 1 | | | | | | 1 | | | 207 | | | | | | | 0.6 | | | | | 0.448 |
| treatment*encapsulation | 1 | | | | | | 1 | | | 207.1 | | | | | | | 1.3 | | | | | 0.254 |
| treatment*sex | 1 | | | | | | 1 | | | 203.8 | | | | | | | 1.4 | | | | | 0.235 |
| treatment*pupal weight | 1 | | | | | | 1 | | | 209.9 | | | | | | | 0.8 | | | | | 0.360 |
| pupal weight | 1 | | | | | | 1 | | | 210.1 | | | | | | | 0.005 | | | | | 0.941 |
| treatment | 1 | | | | | | 1 | | | 208.1 | | | | | | | 0.08 | | | | | 0.774 |
|  |  | | | | | |  | | |  | | | | | | |  | | | | |  |
| **Survival** | | | | | | | | | | | | | | | | | | | | | | |
|  | | | | | | | | | | | | | | | | | | | | | | |
|  | *Nparm* | | | | | | *DF* | | | | | | | | *Wald ChiSquare* | | | | *Prob > ChiSq* | | | |
| treatment*sex | 1 | | | | | | 1 | | | | | | | | 2.9 | | | | 0.091 | | | |
| sex | 1 | | | | | | 1 | | | | | | | | 0.1 | | | | 0.752 | | | |
| treatment | 1 | | | | | | 1 | | | | | | | | 0.2 | | | | 0.632 | | | |
|  |  | | | | | |  | | | | | | | |  | | | |  | | | |

**Figure A1.** Adult survival in the population enclosure. The starting date was set on the day on which all the adults were released in the enclosure (third day of the experiment). Control females and males are shown in black and red solid lines, and food limited females and males are shown in black and red dotted lines, respectively. No significant difference in survival was detected in relation to sex or food limitation treatment (Table A2).
